# Audiological biomarkers of tinnitus in an older Portuguese population

**DOI:** 10.3389/fnagi.2022.933117

**Published:** 2022-08-24

**Authors:** Haúla F. Haider, Diogo Ribeiro, Sara F. Ribeiro, Nuno Trigueiros, Helena Caria, Luís Borrego, Iola Pinto, Ana L. Papoila, Derek J. Hoare, João Paço

**Affiliations:** ^1^ENT Department, CUF Tejo Hospital – NOVA Medical School, Faculty of Medical Sciences, NOVA University Lisbon, Lisbon, Portugal; ^2^Comprehensive Health Research Centre, NOVA Medical School, Faculty of Medical Sciences, Lisbon, Portugal; ^3^NOVA Medical School, Faculty of Medical Sciences, NOVA University Lisbon, Lisbon, Portugal; ^4^ENT Department, Hospital Pedro Híspano, Matosinhos, Portugal; ^5^BTR Unit, Deafness Research Group, BioISI, Faculty of Sciences, University of Lisbon (FCUL), Lisbon, Portugal; ^6^ESS/IPS – Biomedical Sciences Department, School of Health, Polytechnic Institute of Setubal, Setúbal, Portugal; ^7^Department of Immunoallergy, CUF Descobertas Hospital, Lisbon, Portugal; ^8^Instituto Superior de Engenharia de Lisboa, Lisbon, Portugal; ^9^Centro de Matemática e Aplicações, NOVA School of Science and Technology, FCT NOVA, Costa da Caparica, Portugal; ^10^School of Medicine, NIHR Nottingham Biomedical Research Centre, Hearing Sciences, Mental Health and Clinical Neurosciences, University of Nottingham, Nottingham, United Kingdom

**Keywords:** tinnitus, audiological biomarkers, hearing loss, pure tone average, auditory brainstem response, distortion product otoacoustic emissions

## Abstract

Tinnitus is a phantom sound perceived in the absence of external acoustic stimulation. It is described in a variety of ways (e.g., buzzing, ringing, and roaring) and can be a single sound or a combination of different sounds. Our study evaluated associations between audiological parameters and the presence or severity of tinnitus, to improve tinnitus diagnosis, treatment, and prognosis. Our sample included 122 older participants (63 women and 59 men), aged 55–75 years from the Portuguese population, with or without sensory presbycusis and with or without tinnitus. All participants underwent a clinical evaluation through a structured interview, Ear, Nose, and Throat observation, and audiological evaluation (standard and extended audiometry, psychoacoustic tinnitus evaluation, auditory brainstem responses, and distortion product otoacoustic emissions). The Tinnitus Handicap Inventory was used to measure tinnitus symptom severity. Our data confirmed that the odds of developing tinnitus were significantly higher in the presence of noise exposure and hearing loss. Also, participants who had abrupt tinnitus onset and moderate or severe hyperacusis featured higher odds of at least moderate tinnitus. However, it was in the ABR that we obtained the most exciting and promising results, namely, in wave I, which was the common denominator in all findings. The increase in wave I amplitude is a protective factor to the odds of having tinnitus. Concerning the severity of tinnitus, the logistic regression model showed that for each unit of increase in the mean ratio V/I of ABR, the likelihood of having at least moderate tinnitus was 10% higher. Advancing knowledge concerning potential tinnitus audiological biomarkers can be crucial for the adequate diagnosis and treatment of tinnitus.

## Introduction

Tinnitus is a phantom sound perceived in the absence of external acoustic stimulation that can be described in a variety of ways (e.g., buzzing, ringing, and roaring) and can be a single sound or a combination of different sounds (Stouffer and Tyler, 1990; Coles, 1995). It can be perceived in one ear, both ears, or the head, as a constant sound fluctuating in intensity (loudness) or frequency (pitch). Tinnitus is frequently perceived as extremely loud, but when matched with calibrated acoustic signals, is typically within 10 dB of the audiometric threshold (Hall and Haynes, 2001). Tinnitus is categorized as objective or subjective. Objective tinnitus describes a real sound produced by the body that can be heard by an examiner. In contrast, an examiner cannot hear subjective tinnitus. Subjective tinnitus is thought to be caused by abnormal neural activity in the peripheral and/or central auditory system (Møller, 2006).

Tinnitus has a variety of etiological factors and may be associated with other diseases, as it is usually viewed only as a symptom. It often accompanies hearing loss (HL) or hyperacusis, but neither is necessary for its presence (Eggermont and Roberts, 2004; Eggermont, 2013, 2015). Most studies have found that participants with high-pitched tinnitus have HL at high frequencies, and the participants with low-pitched tinnitus (below 1.5 kHz) more frequently have low-frequency HL (König et al., 2006; Moore et al., 2010; Sereda et al., 2011). In a recent study involving participants with presbyacusis and a mean age of 69.75 years (SD = 6.53), the authors found an average pitch of 4,781.3 Hz in men and 3,869.8 Hz in women, considering tinnitus participants (Seimetz et al., 2016).

The causes and pathogenesis of tinnitus remain unclear, and there are no objective audiological or non-audiological tests for the diagnosis of tinnitus. Currently, tinnitus presence and impact are established using self-report and subjective measures, such as questionnaires, e.g., Tinnitus Handicap Inventory (THI) (Newman et al., 1996), or the Tinnitus Reaction Questionnaire (Wilson et al., 1991; Henry et al., 2013; Szczepek et al., 2014).

Psychoacoustic assessment of tinnitus can also be performed. Even though participants with similar psychoacoustic measurements may report very different impacts on their lives, it may be useful to interpret neurophysiological mechanisms of tinnitus.

There are two main theories regarding tinnitus pitch prediction in cases where the tinnitus is accompanied by HL, particularly in sloping configurations. On the one hand, several authors argue that tinnitus pitch should be associated with the audiogram’s edge frequency, corresponding to the boundary between a region of normal or near-normal hearing and a region of more significant HL (Moore et al., 2010; Langers et al., 2012). On the other hand, the most accepted theory supports that perceived tinnitus pitch frequently coincides with frequency regions in which hearing thresholds are most elevated (Norena et al., 2002; Roberts et al., 2008; Tziridis et al., 2022).

Abnormal synchronous neural activity can be identified by specialized clinical tests, namely, auditory-evoked potentials (AEP). Previous studies have used AEP measures to study abnormal neuronal activity in tinnitus participants (Gopal et al., 2004, 2017; Kehrle et al., 2008; Gu et al., 2012; Hsu et al., 2013; Gopal et al., 2017). The most widely used AEP is the auditory brainstem response (ABR), a series of vertex-positive waves that occur within 15 ms of the onset of a click stimulus in human adults.

Differences in ABR traces can be seen depending on the type of stimulus used to evoke the response, type of HL, the degree of HL, and the presence of tinnitus, among others. Concerning the degree of HL and the type of stimulus used, elevated hearing thresholds reduce wave V amplitude to click stimuli, so using tone burst ABR when the tone burst characteristic frequency falls within the frequency region of the HL may provide higher sensitivity (Lewis et al., 2015). According to Serpanos, if more frequency-specific stimuli are used, such as brief tones, in sloping configurations of cochlear HL, more precise information on the relationship between the loudness growth and ABR wave V latency can be obtained (Serpanos, 2004).

Although there is a lack of consensus regarding the use of AEP as a diagnostic tool of tinnitus, mostly because of the lack of homogeneity in participant groups and methodologies, AEP measures may contribute to the clarification of the origin of tinnitus and provide objective diagnostic indicators (Gopal et al., 2017). Furthermore, identifying potential correlations between ABR readings and tinnitus pitch can help formalize tinnitus diagnostic procedures (Pinkl et al., 2017).

Considering the models of pathological enhanced neural synchrony and the potential cortical influence on subcortical tuning functions, it is hypothesized that if there are unique ABR features in tinnitus they will become more pronounced if the ABR parameters are adjusted from click stimuli to tone burst stimuli matched to the tinnitus pitch. However, there are recognized difficulties in tinnitus pitch and loudness matching, and these difficulties occur even in the same individual due to intrinsic or even extrinsic variabilities (Norena et al., 2002; Roberts et al., 2006). Moreover, the association between tinnitus perception and the frequency band power in electroencephalography and magnetoencephalography is not standardized [For a review, see the work of Sedley et al. (2016)].

Given the likely role of cochlear function in the generation of tinnitus, it is essential to assess the inner ear. Otoacoustic emissions (OAE) are sound signals produced by the cochlea and reflect the activity of the outer hair cells (OHC). Through OAE, the cochlear function can be tested in an objective and non-invasive way (Lapsley and Marshall, 2007; Fabijańska et al., 2012). Studies measuring OAE in tinnitus participants have used distortion product otoacoustic emissions (DPOAE) to measure a wide range of primary frequencies (f1 and f2) and levels (L1 and L2) (Fabijańska et al., 2012). Two cochlear processes explain the generation mechanisms of DPOAE. The first is a nonlinear interaction of the primary tones induced by the traveling wave, mainly at the cochlear site in and around the basal region to the f2 location, and the second is a linear coherent reflection of the traveling wave from the location corresponding to the distortion product frequency of 2f1-f2 (Kalluri and Shera, 2001; Fabijańska et al., 2012).

In the literature, there are conflicting results regarding the levels of DPOAE in tinnitus participants. Some studies report decreased DPOAE levels in tinnitus participants compared to controls (Shiomi et al., 1997; Ozimek et al., 2006), whereas others see an increase in DPOAE levels in tinnitus participants (Janssen et al., 1998; Gouveris et al., 2005). If we consider HL, the results become even more complicated. Ami et al. found a significant reduction in the mean baseline DPOAE levels in participants with normal hearing and tinnitus compared to participants with a normal hearing without tinnitus, suggesting that reduced OHC activity would result in tinnitus even before there is a shift in hearing threshold (Ami et al., 2008). However, in the case of HL, findings are reversed; in a group, without tinnitus, there was a markedly reduced mean DPOAE compared to a group with tinnitus. From this, it could be postulated that markedly low levels of cochlear hair cell activity may actually cease the source of aberrant peripheral neural activity in tinnitus. Sztuka et al. (2010) found opposite results, where participants with normal hearing with tinnitus have markedly higher DPOAE amplitudes compared to participants with normal hearing and without tinnitus, suggesting that tinnitus may be caused by increased motility of the OHC induced by decreasing efferent fiber activity, and not by OHC failure.

Identifying reliable audiological biomarkers in participants with tinnitus will allow us to improve the diagnosis, treatment, and prognostic of tinnitus (Ami et al., 2008). The present study aims to identify associations between audiological parameters and the presence of tinnitus, enabling improvement of its diagnosis and treatment. Additionally, variables characterizing these participants will be analyzed, in an attempt to look for associations with the severity of tinnitus.

## Materials and methods

### Participants

The study considered a sample of 122 older participants (63 women and 59 men). According to World Health Organization (WHO), aging is categorized as middle age (45–59 years), elderly (60–74 years), elder (75–90 years), and extreme old age (90 years upward). Since we aimed to study older individuals, we decided that the inclusion criterion would be being in the age group of 55–75 years, which would give us a good appreciation of the aging process regarding tinnitus and related comorbidities. Participants were consecutively recruited from Ear, Nose, and Throat consultations at CUF Infante Santo Hospital from March 2016 to December 2018.

Exclusion criteria were tinnitus from the disease of the outer ear (obliterative exostosis and external otitis), Ménière’s disease, chronic otitis media, otosclerosis, history of ototoxic drug use, exposure to massive noise, history of previous malignancy with chemotherapy, history of autoimmune disorders, neurodegenerative or demyelinating disease, uncompensated medical disorder, or a severe psychiatric disorder. All participants were subjected to immittance audiometry to rule out middle ear pathology (Model: Madsen Zodiac 901, Serial No.:389122).

Additionally, participants unable to comprehend and sign the informed consent or with cognitive impairment were also excluded.

### Clinical evaluation

Data were collected from all participants concerning their personal clinical history (past and present), family history, and audiological assessment, including a tinnitus intensity rating on a scale from 0 to 10 (10 being the loudest possible) (Adamchic et al., 2012). Clinical evaluation included a complete Ear, Nose, and Throat examination. Epidemiologic data (demographics, previous and present diseases, toxicological habits, and noise exposure) were collected using a structured interview.

### Audiological assessment

#### Tonal audiometry

Pure tone audiometry (air and bone) was conducted to evaluate hearing thresholds according to ISO 8253 and 389. Standard tonal and extended high-frequency audiometry (250 Hz to 16K kHz) was performed in a soundproof booth employing an Interacoustics,^®^ Assens, Denmark audiometer (Model: AC40, Serial No.: 98 019 046) and TDH39/HDA300 headphones fitted with noise-excluding headset ME70 and bone conductor B-71 were used.

The category of HL was defined according to the recommendations of the Bureau International d’Audiophonologie as follows: normal or subnormal hearing (below 20 dB), mild HL (21–40 dB), moderate HL (41–70 dB), severe HL (71–90 dB), very severe HL (91–119 dB), or total HL-cophosis (over 120 dB) (Bureau International d’Audiophonologie [BIAP], 1996). Pure tone average (PTA) was taken as the average threshold across 500 Hz, 1,000 Hz, 2,000 Hz, and 4,000 Hz. Frequencies not heard were evaluated as 120 dB threshold. “High-frequency” pure tone average (“HF” PTA) was calculated as the average thresholds across 2, 4, and 8 kHz (Newman et al., 2012). For both PTA and “HF” PTA, the averages were calculated with both ears.

The presence or absence of presbyacusis and the presence of tinnitus were recorded. Presbycusis was defined as bilateral sensorineural deafness in a downslope audiometric pattern, above 1,000 Hz, with poor speech discrimination (Speech Recognition Threshold >40 dB SPL and 100% discrimination to 60 dB or worse) (Schuknecht and Gacek, 1993).

### Tinnitus assessment

Several tests for measurement and evaluation of tinnitus were performed on all the participants having this complaint.

The same experimenter performed all the audiological tests in a standardized protocol.

#### Psychoacoustic tinnitus evaluation

Tinnitus evaluation was performed after audiometric testing in a soundproof booth using an Interacoustics,^®^ Assens, Denmark audiometer (Model: AC40, Serial No.: 98 019 046) and TDH39/HDA300 headphones fitted with noise-excluding headset ME70. First, it was established whether the tinnitus percept was more similar to a pure tone or a narrow-band noise. Both sounds were presented to the participant who was asked which of the two had the most resemblance to their tinnitus.

Estimation of tinnitus frequency was then performed using frequencies from 125 to 16 kHz (pure tones or narrow-band noise centered on the same frequencies). The procedure for determining tinnitus pitch was a forced choice between two presented stimuli. Stimuli were presented to the participant who identified which most closely resembled their tinnitus. The test continued until a correspondence between the tinnitus and the presented stimulus was found. For the estimation of tinnitus loudness (intensity), the determined frequency (from the previous step) was presented at an intensity similar to the individual’s hearing threshold and gradually increased (5 dB steps) until it reached the closest matching to the participant’s tinnitus percept.

#### Loudness discomfort levels

The collection of the discomfort thresholds was performed for each ear individually on the frequencies tested in the tonal audiogram and the frequency at which the tinnitus was identified using pure tones, beginning at the hearing threshold, using an ascendant process with 5 dB increments. The patient was instructed to signal when the sound becomes uncomfortable, not only loud but also uncomfortable. Three tests should be carried out to investigate the thresholds to ensure the test’s reliability (Goldstein and Shulman, 1996).

The difference between the auditory threshold and the discomfort thresholds gave the dynamic auditory field (Goldstein and Shulman, 1996). Once this was determined, the presence or absence of hyperacusis was evaluated.

#### Feldmann masking curves or minimum masking levels

This test was performed at the frequencies where standard tonal audiometry was tested, using narrow-band noises or pure tones (where narrow-band noises did not mask tinnitus). The sound was presented in 5 dB steps (1–2 s stimulation), from hearing thresholds, until the participant reported that they could no longer hear their tinnitus. According to the spatial relationship of the resulting curves from hearing thresholds and tinnitus masking, Feldmann’s masking curves were categorized as follows: 1, Convergent; 2, Divergent; 3, Congruent; 4 Distant; and 5, Persistent (Goldstein and Shulman, 1997).

#### Residual inhibition

Residual inhibition or residual excitation was tested by presenting participants with a narrow-band noise centered at their tinnitus pitch, at 10 dB above the tinnitus loudness, for 1 min. RI was categorized as follows: 1, complete (tinnitus is not audible); 2, partial (tinnitus became quieter); 3, negative (no change at tinnitus percept); and 4, “rebound” effect (tinnitus became louder). In categories 1, 2, and 4, we measured the duration of time that tinnitus was abolished or diminished in seconds or minutes, the time that it takes for the tinnitus percept to come back to basal characteristics in terms of loudness (Coles and Hallam, 1987; Goldstein and Shulman, 1997).

### Tinnitus handicap inventory

Self-reported tinnitus severity was measured using the Portuguese validated version of the THI (Oliveira and Meneses, 2008). This inventory consists of 25 questions related to tinnitus, with “Yes,” “Sometimes,” and “No” as possible responses, corresponding to scores of 4, 2, and 0, respectively, giving a total score between 0 and 100. This questionnaire consists of three sub-scales: functional (11 items, contributing 0–44 for the final result), emotional (9 items, contributing 0–36 for the final result), and catastrophic (5 items, contributing 0–20 for the final result). Severity is interpreted according to the total score, where 0–16 indicates slight or no handicap (Grade 1), 18–36 indicates mild handicap (Grade 2), 38–56 indicates moderate handicap (Grade 3), 58–76 indicates severe handicap (Grade 4), and 78–100 indicates catastrophic handicap (Grade 5). We have used the cutoff THI >37 for statistical comparison purposes, in agreement with the European Tinnitus Guidelines (Cima et al., 2019). In order to better interpret the results, we refer to the group that covers moderate, severe, and catastrophic severity as “at least moderate.”

### Auditory brainstem response

Auditory brainstem response examination was performed in a soundproofed electrically insulated room. The participant was placed in a comfortable position in order to ensure proper relaxation of cervical muscles. The Vivosonic audiometer system (Model: IntegrityTM V500, Serial No. IP0960) was used to collect ABR and determine electrophysiological thresholds. The earphones used were the ER-3A, calibrated according to ANSI S3.6-1996, and a 4,000 Hz tone burst was used to evoke ABR, calibrated in decibel peak equivalent to the sound pressure level (Jiang, 1998). We used an alternating split polarity with a stimulus rate of 27.7 stimuli/s, a high pass filter cutoff frequency at 30 Hz, a low pass filter cutoff frequency at 1,500 Hz, a high pass filter roll of 12 dB/Octave, a low pass filter roll off at 24 dB/Octave, notch filter off, a Blackman windowing, and a rise-plateau-fall of 2-0-2. The non-inverting electrode was placed according to the 10–20 system at the frontal upper forehead (Fz) and the inverting electrode at the mastoid (M1,2) at the examining side (Jasper, 1958). The neutral electrode was placed at the frontal lower forehead (Fzd) region. The monoaural parameters evaluated were the absolute latencies for waves I, III, and V, interwave (interpeak) latency interval (IWI) for waves I-III, III-V, and I-V, amplitude wave I and V, and V/I amplitude ratio. For all variables analyzed, an average for both ears was used.

### Distortion product otoacoustic emissions

The distortion product otoacoustic emissions were performed using a Vivosonic audiometer system in a soundproofed room. We tested the DPOAE for the frequencies of 500, 750, 1,000, 1,500, 2,000, 2,500, 3,000, 3,200, 3,500, 4,000, 4,500, 5,000, 5,500, 6,000, 7,000, and 8,000 Hz, with a 1.22 F2/F1 ratio and with an intensity of 65 dB SPL and 55 dB SPL for L1 and L2, respectively. The presence of OAE was considered when the signal-to-noise ratio was equal to or above 6 dB. For all variables analyzed, an average for both ears was used.

### Statistical analysis

An exploratory analysis of all registered variables was carried out initially, followed by a data modeling phase. Categorical data were presented as frequencies and percentages, and continuous variables as median and inter-quartile range (25th percentile and 75th percentile), as they presented asymmetric distributions and deviations from normality.

The nonparametric chi-square test or Fisher’s exact test was used for qualitative variables, and for the continuous variables, the Mann–Whitney U-test was applied.

When clinically relevant, some of the variables were recorded. Additionally, the self-reported tinnitus severity score was recoded into a binary variable: Grades 1, 2, and 3 (Low THI score) vs. Grades 4 and 5 (High THI score).

To assess the association between the presence of tinnitus or tinnitus severity and the demographic and audiological variables, univariable logistic regression analyses were performed. Odds ratios estimates $\left(\hat{O\,R}\right)$ and corresponding 95% confidence intervals (95% CI) were thus obtained. Logistic regression logit linearity assumption was assessed using the Box-Tidwell test (Box and Tidwell, 1962).

Additionally, to evaluate the discriminative ability (tinnitus vs. non-tinnitus groups) of some of the audiological parameters, the area under the ROC (receiver operating characteristic) curve (AUC) was reported.

The level of significance α = 0.05 was considered. Statistical analyses were performed in SPSS (IBM Corp. Released 2013. IBM SPSS Statistics for Windows, Version 22.0. Armonk, NY: IBM Corp.).

## Results

### Participant’s demographics and comorbidities

In this study, 122 participants were recruited with a median age of 63.0 (59.0; 68.3) years. In women (*n* = 63, 51.6%), we obtained a median age of 63.0 (59.0; 69.0) years, while in men (*n* = 59, 48.4%), the median age was 63.0 (59.0; 68.0) years.

Concerning comorbidities, mumps was present in 56% of the tinnitus group and 40.0% of the group without tinnitus. Also, 53.3% of the population with tinnitus and 26.7% without tinnitus had HL. For further details, please see Supplementary material 1.

### Audiological assessment

The sample was naturally divided into four subgroups (Table 1): the subgroup without HL at standard frequencies and without tinnitus (Subgroup 1), without HL at standard frequencies but presenting tinnitus (Subgroup 2), with HL but without tinnitus (Subgroup 3), and participants with both HL and tinnitus (Subgroup 4). These groups allowed comparisons between the presence (Subgroup 2 + Subgroup 4) and absence (Subgroup 1 + Subgroup 3) of tinnitus.

**TABLE 1:** Distribution of the participants of the sample by four subgroups.

Subgroup	Audiological characteristic	Gender (n)		n (%)	Age* years
		Male	Female		
1	PTA ≤ 20 without Tinnitus	7	15	22 (18.0)	63.0 (59.0; 68.3)
2	PTA ≤ 20 with Tinnitus	15	27	42 (34.4)	63.0 (59.0; 68.3)
3	PTA > 20 without Tinnitus	6	2	8 (6.6)	63.0 (59.0; 68.3)
4	PTA > 20 with Tinnitus	31	19	50 (41.0)	63.0 (59.0; 68.3)
**Total**		59	63	122	

PTA, Pure tone average. *Data are summarized as median (25th percentile; 75th percentile).

Comparing the non-tinnitus participants versus tinnitus participants, PTA and “HF” PTA were statistically higher in those with tinnitus (Table 2).

**TABLE 2:** PTA and “HF” PTA according to tinnitus presence.

Variables	All participants (*n* = 122)	With tinnitus (*n* = 92; 75.4%)	Without tinnitus (*n* = 30; 24.6%)	*P*-value*
Mean PTA (dB)	20.0 (14.8; 28.3)	21.6 (16.4; 29.4)	16.9 (12.3; 21.4)	0.009
Mean “HF” PTA (dB)	35.0 (23.3; 47.7)	37.9 (28.8; 48.3)	25.4 (18.3; 34.2)	0.001

Data are summarized as median (25th percentile; 75th percentile); PTA, Pure tone average, “HF” PTA, “High frequency” pure-tone average; *p-values were obtained by univariable logistic regression models.

### Auditory brainstem response

**FIGURE 1:**
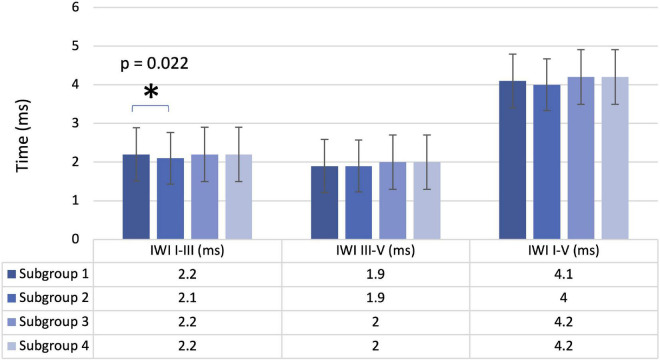
Interpeak latency intervals I-III, III-V, and I-V for the four subgroups. Mean values with error bars: column height represents the mean, and the bars of each column show the standard deviation.

When comparing ABR across the four subgroups (Table 3), significant differences in I-III intervals were found between Subgroup 1 and Subgroup 2 (*p* = 0.022) (Figure 1). In subgroup 1, the time it takes for the stimulus to travel, on average, through the interval between wave I and wave III is 2.2 ms (2.2; 2.4), while in subgroup II, there is a decrease in this same interval, that is, 2.1 ms (2.0; 2.2).

**TABLE 3:** Comparison of auditory brainstem response in the four subgroups.

Auditory brainstem response	All participants (*n* = 122)	PTA ≤ 20 without tinnitus (*n* = 22; 18.0%)	PTA ≤ 20 with tinnitus (*n* = 42; 34.4%)	PTA > 20 without tinnitus (*n* = 8; 6.6%)	PTA > 20 with tinnitus (*n* = 50; 41.0%)	*P*- value*
Wave I Latency (ms)	2.0 (1.9; 2.1)	2.0 (1.9; 2.1)	2.1 (1.9; 2.2)	2.0 (1.9; 2.2)	2.0 (1.9; 2.2)	0.647
Wave III Latency (ms)	4.2 (4.0; 4.4)	4.1 (3.9; 4.3)	4.2 (4.0; 4.3)	4.1 (3.3; 4.3)	4.3 (4.1; 4.5)	0.007^(a)^
Wave V Latency (ms)	6.1 (6.0; 6.3)	6.1 (5.8; 6.2)	6.1 (5.9; 6.2)	6.0 (5.4; 6.4)	6.3 (6.1; 6.4)	0.001^(b)^
IWI I-III (ms)	2.2 (2.1; 2.3)	2.2 (2.2; 2.4)	2.1 (2.0; 2.2)	2.2 (2.0; 2.4)	2.2 (2.1; 2.4)	0.011^(c)^
IWI III-V (ms)	1.9 (1.8; 2.0)	1.9 (1.8; 2.0)	1.9 (1.8; 2.0)	2.0 (1.9; 2.1)	2.0 (1.8; 2.1)	0.197
IWI I-V (ms)	4.1 (4.0; 4.3)	4.1 (4.0; 4.3)	4.0 (3.1; 4.2)	4.2 (4.0; 4.4)	4.2 (4.1; 4.3)	0.003^(d)^
Amplitude wave I (μV)	0.07 (0.05; 0.11)	0.09 (0.05; 0.15)	0.09 (0.05; 0.11)	0.07 (0.05; 0.11)	0.06 (0.03; 0.09)	0.007^(e)^
Amplitude wave V (μV)	0.2 (0.1; 0.3)	0.22 (0.15; 0.30)	0.24 (0.16; 0.29)	0.15 (0.11; 0.19)	0.14 (0.08; 0.18)	< 0.001 ^(f)^
V/I amplitude ratio (μV)	2.9 (1.8; 6.6)	2.3 (1.7; 3.8)	3.2 (2.3; 7.7)	2.5 (2.0; 7.7)	3.0 (1.8; 7.8)	0.358

Data are summarized as median (25th percentile; 75th percentile); IWI, Interwave latency interval, PTA, Pure tone average; *p-values were obtained by Kruskal–Wallis test.

(a) p = 0.031 for the groups PTA ≤ 20 with tinnitus and PTA > 20 with tinnitus.

(b) p = 0.003 for the groups PTA ≤ 20 with tinnitus and PTA > 20 with tinnitus; p = 0.016 for the groups and PTA > 20 with tinnitus and PTA ≤ 20 without tinnitus.

(c) p = 0.036 for the groups PTA ≤ 20 with tinnitus and PTA > 20 with tinnitus; p = 0.022 for the groups PTA ≤ 20 with tinnitus and PTA ≤ 20 without tinnitus. (d) p = 0.002 for the groups PTA ≤ 20 with tinnitus and PTA > 20 with tinnitus.

(e) p = 0.012 for the groups PTA ≤ 20 without tinnitus and PTA > 20 with tinnitus.

(f) p < 0.001 for the groups PTA ≤ 20 with tinnitus and PTA > 20 with tinnitus; p < 0.001 for the groups PTA > 20 with tinnitus and PTA ≤ 20 without tinnitus.

**FIGURE 2:**
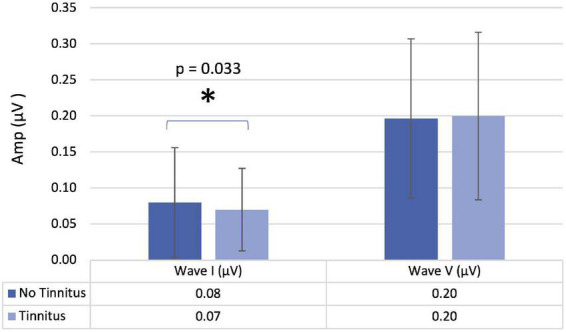
Amplitude of waves I and V in participants with and without tinnitus. Mean values with error bars: column height represents the mean, and the bars of each column show the standard deviation.

When comparing participants with and without tinnitus (Table 4), there was a significant difference in the amplitude of wave I (*p* = 0.033) (Figure 2). When analyzing these results in more detail, we found that in the participants with tinnitus, the amplitude of wave I was 0.07 μV (0.04; 0.10), while in the group without tinnitus, we verified a significant increase to 0.08 μV (0.05; 0.15).

**TABLE 4:** Comparison of auditory brainstem response between participants with and without tinnitus.

Auditory brainstem response	All participants (*n* = 122)	With tinnitus (*n* = 92; 75.4%)	Without tinnitus (*n* = 30; 24.6%)	*P*-value*
Wave I Latency (ms)	2.0 (1.9; 2.1)	2.0 (1.9; 2.2)	2.0 (1.9; 2.1)	0.115
Wave III Latency (ms)	4.2 (4.0; 4.4)	4.2 (4.1; 4.4)	4.1 (3.5; 4.3)	0.695
Wave V Latency (ms)	6.1 (6.0; 6.3)	6.2 (6.0; 6.3)	6.1 (5.5; 6.3)	0.968
IWI I-III (ms)	2.2 (2.1; 2.3)	2.2 (2.1; 2.3)	2.2 (2.1; 2.4)	0.197
IWI III-V (ms)	1.9 (1.8; 2.0)	1.9 (1.8; 2.0)	2.0 (1.9; 2.0)	0.597
IWI I-V (ms)	4.1 (4.0; 4.3)	4.1 (4.0; 4.3)	4.2 (4.0; 4.3)	0.139
Amplitude wave I (μV)	0.07 (0.05; 0.11)	0.07 (0.04; 0.10)	0.08 (0.05; 0.15)	0.033
Amplitude wave V (μV)	0.2 (0.1; 0.3)	0.2 (0.1; 0.2)	0.2 (0.1; 0.3)	0.178
V/I amplitude ratio (μV)	2.9 (1.8; 6.6)	3.2 (1.9; 7.7)	2.4 (1.7; 4.3)	0.340

Data are summarized as median (25th percentile; 75th percentile); IWI, Interwave latency interval; *p-values were obtained by the Mann–Whitney U test.

### Distortion product otoacoustic emissions

**FIGURE 3:**
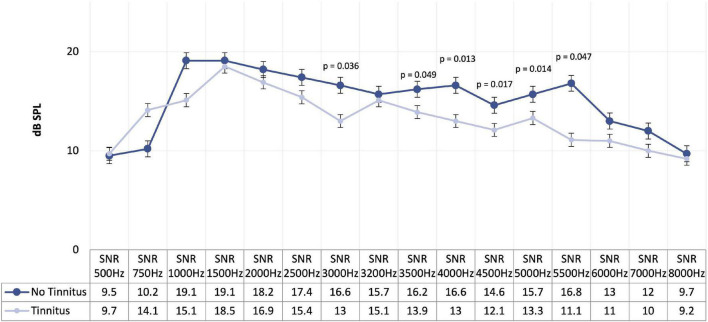
Mean of distortion product otoacoustic emissions in participants with and without tinnitus.

When comparing the four subgroups regarding DPOAE, no significant differences were found. On the other hand, when we compared participants with and without tinnitus, we identified significant differences for the mean DPOAE values between 500 and 8,000 Hz (*p* = 0.014), and for the 3,500 Hz (*p* = 0.049), 4,000 Hz (*p* = 0.013), 4,500 Hz (*p* = 0.017), 5,500 Hz (*p* = 0.014), and 6,000 Hz (*p* = 0.047) (Figure 3 and Table 5). In more detail, we see a decrease in DPOAE for the frequencies of 3,000, 3,500, 4,000, 4,500, 5,500, and 6,000 Hz in the group with tinnitus compared to the group without tinnitus.

**TABLE 5:** Comparison of distortion product otoacoustic emissions in participants with and without tinnitus.

Distortion product otoacoustic emissions (Hz)	All participants (*n* = 122)	With tinnitus (*n* = 92; 75.4%)	Without tinnitus (*n* = 30; 24.6%)	*P*-value*
Mean 500–8000	122	*n* = 92 13.0 (11.2; 16.1)	*n* = 30 15.0 (12.5; 17.4)	0.014
500	69	n = 54 9.7 (7.9; 12.4)	n = 15 9.5 (7.7;19.8)	0.432
750	104	*n* = 77 14.1 (10.0; 17.7)	*n* = 27 10.2 (8.4; 17.9)	0.247
1000	112	*n* = 84 15.1 (11.9; 19.8)	*n* = 28 19.1 (13.1; 21.3)	0.052
1500	117	*n* = 87 18.5 (13.5; 20.2)	*n* = 30 19.1 (15.1; 21.0)	0.394
2000	115	*n* = 86 16.9 (12.1; 20.0)	*n* = 29 18.2 (14.2; 19.6)	0.515
2500	110	*n* = 82 15.4 (11.6; 19.4)	*n* = 28 17.4 (12.7; 19.8)	0.402
3000	115	*n* = 87 13.0 (9.8; 18.0)	*n* = 28 16.6 (12.9; 18.9)	0.036
3200	105	*n* = 78 15.1 (11.4;19.0)	*n* = 27 15.7 (11.7; 18.9)	0.703
3500	107	*n* = 80 13.9 (9.8; 18.0)	*n* = 27 16.2 (13.2; 19.8)	0.049
4000	102	n = 76 13.0 (10.0;17.7)	*n* = 26 16.6 (12.8; 19.8)	0.013
4500	101	*n* = 73 12.1 (9.7; 17.1)	*n* = 28 14.6 (12.3; 19.5)	0.017
5000	100	*n* = 73 13.3 (10.3; 17.7)	*n* = 27 15.7 (12.0; 19.7)	0.144
5500	90	*n* = 68 11.1 (9.3; 16.2)	*n* = 22 16.8 (12.4; 18.7)	0.014
6000	100	*n* = 72 11.0 (8.7; 13.4)	*n* = 28 13.0 (10.7; 17.0)	0.047
7000	103	*n* = 80 10.0 (8.1; 13.0)	*n* = 23 12.0 (9.3; 14.8)	0.091
8000	90	*n* = 65 9.2 (7.9; 11.7)	*n* = 25 9.7 (8.6; 11.1)	0.428

Data are summarized as median (25th percentile; 75th percentile). Mean = mean of DPOAE frequencies from 500 to 8,000 Hz. *p-values were obtained by the Mann–Whitney U test.

### Tinnitus group evaluation

The clinical characteristics of participants with tinnitus (*n* = 92) are presented in Table 6. The median duration of tinnitus was 5.0 (2.0; 10.0) years, with a rather mild median intensity of 3.0 (2.0; 4.0) on a scale of 1–10. Tinnitus was constant for most participants (88.9%), while onset was gradual for 71.4% and abrupt for 28.6%. In many participants, tinnitus worsened in situations where they were nervous (59.3%). Finally, 50.6% of the participants with tinnitus reported reduced noise tolerance.

**TABLE 6:** Clinical characterization of tinnitus sample.

Clinical variables	Participants with tinnitus (*n* = 92)
Tinnitus duration (in years)	5.0 (2.0; 10.0)
Intensity of tinnitus (scale 1–10)	3.0 (2.0; 4.0)
**Manifestation of tinnitus** (*n* = 90)	
Constant	80 (88.9)
Intermittent	6 (6.7)
Pulsatile	4 (4.4)
**How did tinnitus begin?** (*n* = 63)	
Gradual	45 (71.4)
Abrupt	18 (28.6)
**Does tinnitus get worse when you’re nervous?** (*n* = 91)	
Yes	54 (59.3)
No	37 (40.7)
**Lower noise tolerance** (*n* = 89)	
Yes	45 (50.6)
No	44 (49.4)
**Familiar history with tinnitus**	
Yes	25 (29.4)
**Dizziness**	
Yes	35 (41.2)
**With deafness**	
Yes	49 (53.3)
**Exposure to noise** (*n* = 91)	
Non exposed	56 (61.5)
Exposed without protection	31 (34.1)
Exposed with protection	4 (4.4)

Continuous variables are summarized as median (25th percentile; 75th percentile) and categorical variables as n (%).

Psychoacoustic estimates of tinnitus are given in Table 7. Percentile frequencies matched to tinnitus pitch were 2,000 Hz (25th percentile) and 8,000 Hz (75th percentile), with a median of 4,000 Hz, while for loudness, a median of 0 dB (25th percentile = 0 dB and 75th percentile = 5 dB) was obtained. Our sample was characterized by more than half with central location (52.4%) and pure tone type of tinnitus (59.0%). Concerning Feldmann’s curve, the convergent (47.6%) and distant types (29.8%) were the most frequent, while in the residual inhibition, the negative (43.9%) and partial (36.6%) types characterize the majority of the sample.

**TABLE 7:** Psychoacoustic tinnitus assessment.

Audiological measurements	Participants with tinnitus (*n* = 92)
Pitch (*n* = 83)	4000 Hz (2000 Hz; 8000 Hz)
Loudness (*n* = 83)	0 dB (0 dB; 5.0 dB)
**Laterality** (*n* = 84)	
Central	44 (52.4)
Right	15 (17.9)
Left	25 (29.8)
**Type** (*n* = 83)	
Pure tone	49 (59.0)
Narrow band noise	34 (41.0)
**Feldmann’s curve** (*n* = 84)	
Congruent	17 (20.2)
Convergent	40 (47.6)
Divergent	1 (1.2)
Distant	25 (29.8)
Persistent	1 (1.2)
**Residual inhibition** (*n* = 82)	
Negative	36 (43.9)
Partial	30 (36.6)
Complete	13 (15.9)
Rebound effect	3 (3.7)

Data are summarized as n (%).

Tinnitus severity was evaluated using THI scores. Thirty-eight participants had a mild handicap (41.3%), followed by 22 with a moderate handicap (23.9%), 17 with slight or no handicap (18.5%), severe handicap in 14 participants (15.2%), and finally, 1 participant had a catastrophic handicap score.

### Noise exposure and distortion product otoacoustic emissions

Table 8 presents the results according to noise exposure in participants with tinnitus. The comparison between the groups with/without noise exposure revealed significant differences in DPOAE.

**TABLE 8:** DPOAE results in participants with tinnitus, according to noise exposure conditions.

Distortion product otoacoustic emissions*(n = 91)*	Submitted to noise exposure (*n* = 35;38.5)	Non-submitted to noise exposure (*n* = 56;61.5)	*P*-value*
Mean 500–8000	12.2 (10.7; 13.3)	14.2 (11.5; 17.1)	0.040

Data are summarized as median (25th percentile; 75th percentile); Mean = mean of DPOAE frequencies from 500 to 8,000 Hz. *p-value was obtained by the Mann–Whitney U test.

### Analyzing the data

Although several variables were identified in the univariable study as potential candidates for the multivariable models of both “presence of tinnitus” and “tinnitus severity” outcomes, no multiple models were obtained because all of those identified variables became statistically non-significant when simultaneously considered.

#### Analyzing the data according to the presence of tinnitus

Six univariable logistic regression analyses were performed for the presence or absence of tinnitus, and the results are presented in Table 9.

**TABLE 9:** Univariable analysis: logistic regression model for the presence of tinnitus.

Variable	$\hat{\text{OR}}$	*P*-value	(95%CI)
Noise exposure	3.96	0.036	(1.09, 14.36)
Mean PTA	1.08	0.009	(1.02, 1.14)
Hearing Loss	3.87	0.014	(1.32, 11.39)
Mean “HF” PTA	1.07	0.001	(1.03, 1.11)
DPOAE mean 500–8000	0.86	0.023	(0.75, 1.11)
Amplitude wave I	0.404^(1)^	0.016	(0.193, 0.844)

^(1)^ odds of having tinnitus for every 10 units of increase of the amplitude wave I; CI confidence interval; PTA = Pure tone average; “HF” PTA = “High frequency” pure-tone average; DPOAE = Distortion product otoacoustic emissions; Mean = mean of DPOAE frequencies from 500 to 8,000 Hz.

From this analysis, we found several variables that were associated with tinnitus. Noise exposure (*p* = 0.036), mean PTA thresholds (*p* = 0.009), HL (*p* = 0.014), and mean “HF” PTA thresholds (*p* = 0.001) increased the odds of tinnitus. However, some other variables represented lower odds of having tinnitus, including, the mean DPOAE between 500 and 800 Hz (*p* = 0.023) and the amplitude of ABR wave I (*p* = 0.016). HL was highly associated with tinnitus; “HF” PTA attained an AUC = 0.72, with 95% CI:0.61, 0.83.

#### Analyzing the data according to the severity of tinnitus

A univariable analysis considering the severity of tinnitus as the outcome variable was performed. Two subgroups were considered, lower THI (slight or no handicap and mild handicap) and higher THI scores (moderate, severe, or catastrophic handicap). Only the significant results pertaining to tinnitus severity are presented in Table 10. These were tinnitus onset (*p* = 0.017), hyperacusis (*p* = 0.030), and residual inhibition (*p* = 0.035).

**TABLE 10:** Univariable analysis: patient characteristics by group (high versus low THI score).

Variables	All participants (*n* = 92)	Low THI score (*n* = 55; 45.1%)	High THI score (*n* = 37; 30.3%)	*P*-value
**Tinnitus onset** (*n* = 63)				
Gradual	45 (71.4)	32 (82.1)	13 (54.2)	0.017^(1)^
Abrupt	18 (28.6)	7 (17.9)	11 (45.8)	
**Hyperacusis** (*n* = 85)				
Negative	66 (77.6)	39 (76.5)	27 (79.4)	0.03^(2)^
Moderate	5 (5.9)	2 (3.9)	3 (8.8)	
Light	11 (12.9)	10 (19.6)	1 (2.9)	
Severe	3 (3.5)	0 (0)	3 (8.8)	
**Residual inhibition** (*n* = 82)				
Negative	36 (43.9)	23 (46.9)	13 (39.4)	0.034^(2)^
Partial	30 (36.6)	13 (26.5)	17 (51.5)	
Complete	13 (15.9)	11 (22.4)	2(6.1)	
Rebound Effect	3 (3.7)	2 (4.1)	1 (3.0)	

^(1)^ Chi-square test p-value; ^(2)^ Fisher’s exact test p-values.

Still considering participants’ characteristics (Table 11), participants with abrupt tinnitus onset were around four times more likely to have at least moderate tinnitus$\left(\hat{O\,R}=3.87,p=0.021,95\%\,C\,I=1.23-12.17\right)$. Participants with moderate or severe hyperacusis had five times higher odds of having at least moderate tinnitus$\left(\hat{O\,R}=5.25,p=0.051,95\%\,C\,I=0.99-27.79\right)$.

**TABLE 11:** Univariable analysis logistic regression model: tinnitus characteristics by group (higher versus lower THI score).

Variables	$\hat{\text{OR}}$ (95 CI)	*P*-value
**Tinnitus appearance**	3.87 (1.23, 12.17)	*p* = 0.021
Gradual		
Abrupt		
**Hyperacusis**	5.25^(1)^ (0.99, 27.79)	*p* = 0.051
Negative + Light		
Moderate + Severe		

$\left(\hat{O\,R}\right)$ odds ratio estimate. ^(1)^ reference category is light or negative hyperacusis.

Concerning ABR evaluation, the logistic regression model showed that for each unit of increase in the mean ratio V/I of ABR, the probability of having at least moderate tinnitus was 10% higher $(\hat{O\,R}=1.10,p=0.046,CI=1.00-1.21$).

## Discussion

This study identifies associations between audiological parameters and the presence of tinnitus. Additionally, variables characterizing these participants were analyzed, and associations with tinnitus severity were identified.

We found statistically significant differences for both the mean PTA thresholds and “HF” PTA thresholds when we compared participants with and without tinnitus. Thus, there is a possible association between the development of HL and the appearance of tinnitus. These results are in agreement with the literature where it has been hypothesized that tinnitus is an epiphenomenon of a neuronal process to attempt normalizing impaired hearing thresholds, that is, a central compensation for peripheral damage (Gollnast et al., 2017). The age range of our study population (55–75 years), most of them presenting presbycusis (sloping configurations), certainly explains higher hearing thresholds at higher frequencies, but thresholds were notably higher in tinnitus participants than those without tinnitus. Gollnast et al. (2017) when comparing tinnitus participants with non-tinnitus participants, verified that in adult participants, hearing thresholds were lower in the low-frequency range, while it was higher at high frequencies in the group of tinnitus participants. Our data also confirm that the odds of having tinnitus were significantly higher in the presence of HL and noise exposure.

Regarding the ABR, one of the findings was the reduction of the amplitude in wave I in tinnitus participants. The reduction of the wave I amplitude is in accordance with the published studies on tinnitus participants (Attias et al., 1993; Schaette and McAlpine, 2011; Gu et al., 2012). From another perspective, the increase in wave I amplitude is a protective factor to the odds of having tinnitus. There are several explanations for this reduced amplitude in wave I, particularly involving changes in the inner hair cells and or auditory nerve fibers (ANFs). Concerning inner hair cells, there may be a diffuse loss of the sensory epithelium, higher in tinnitus participants, which results in a lowered wave I amplitude (Gu et al., 2012). In another model, the inner hair cells are equally intact in both tinnitus and non-tinnitus participants, but in one of them, there is a diffuse loss of the ANFs, while in the other, the ANFs remain intact (Le Prell et al., 2003, 2005; Gu et al., 2012). Another scenario is that ANFs are equally intact, and the reduction of the wave I amplitude is due to the reduced excitability of ANFs *via* lateral olivocochlear efferent, which terminates on their endings, or there is a diffuse loss of ANFs sufficient to manifest a reduction in mean wave I amplitude. Concerning the severity of tinnitus, the logistic regression model showed that for each unit of increase in the mean ratio V/I of ABR, the likelihood of having at least moderate tinnitus was 10% higher. Since no statistically significant differences were found in the amplitude of wave V, we can infer that this finding is exclusively due to the values of the amplitude of wave I, thus also corroborating the various possibilities described above.

Another finding regarding the ABR is the statistical difference concerning the interpeak latency I-III when comparing Subgroup 1 (no HL or tinnitus) and Subgroup 2 (tinnitus but no HL). We can see a diminished interval interpeak I-III in the group with normal hearing with tinnitus. Interestingly, we did not find similar results in our literature review. However, although we did not find significant differences in absolute latency of wave I in our sample when we compared both subgroups, in Subgroup 2, wave I started later than in Subgroup 1. This could explain the difference in the interpeak latencies I-III when we compared both groups, since, according to several authors, in tinnitus participants, wave I has a significant prolongation (Ikner and Hassen, 1990; Lemaire and Beutter, 1995; Rosenhall and Axelsson, 1995; Kehrle et al., 2008). It has been assumed that it signals a peripheral lesion in the auditory system (Rosenhall and Axelsson, 1995; Kehrle et al., 2008). Lemaire and Beutter (1995) found similar results in tinnitus participants and suggested that this modification is due to a dysfunction of the nucleus tegmenti, which is part of the efferent system. Future research should be performed in this direction in order to clarify this finding.

When we compared the four subgroups, considering HL and tinnitus, no significant differences were found in the levels of the DPOAE. However, when we compared participants without tinnitus and tinnitus, not considering the presence or absence of HL, we found significant differences (Figure 3 and Table 5). One of the differences found refers to the mean of the DPOAE between 500 and 8,000 Hz. This finding agrees with the results reported by Shiomi et al. (1997) and Ozimek et al. (2006), which points us to conclude that the observed differences are specific to the OHC functions instead of the nonspecific non-linearity of the basilar membrane system. The most exciting results were obtained when we analyzed each of the frequencies separately; we found statistically significant differences for the high frequencies, namely, for the frequencies of 3,000, 3,500, 4,000, 4,500, 5,500, and 6,000 Hz. Based on this finding, we can state that frequencies that presented statistically significant differences were the frequencies where the perceived tinnitus pitch coincided and the frequency regions in which hearing thresholds were found to be most elevated (Table 7). However, information regarding the relationship between dominant tinnitus pitch and DPOAE parameters is limited.

When we analyzed these findings in more detail, we noticed a decrease in DPOAE in the group with tinnitus compared to the group without tinnitus for all the frequencies where the results were statistically significant. According to Ozimek et al. (2006); Granjeiro et al. (2008), and Sereda et al. (2015), the decrease in DPOAE suggests that cochlear dysfunction is involved in developing this condition, particularly at higher frequencies. On the other hand, not verified in our study, several studies point to an increase in DPOAE levels in tinnitus participants, which indicates that the tinnitus might be generated by the increase in the motility of the OHC, induced by decreasing efferent fiber activity, and not by OHC failure (Janssen et al., 1998; Gouveris et al., 2005; Sztuka et al., 2010). When we add another variable of noise exposure to the DPOAE, we saw a statistically significant decrease in the values of DPOAE in participants with a history of noise exposure. In fact, this was a protective variable, and when it was higher, the odds of having tinnitus diminished. These results are in accordance with those reported by Sindhusake et al. (2003).

In the participants of our sample, tinnitus frequency (pitch) ranged from 2,000 to 8,000 Hz, with 4,000 Hz being the most often found. This could be explained by the expected localization of the tinnitus pitch in the “edge” frequencies or within the lowest regions in participants presenting both HL, particularly sloping configurations as observed in our sample, and tinnitus (Norena et al., 2002; Roberts et al., 2008; Moore et al., 2010; Langers et al., 2012). The tinnitus loudness in our sample varied between 0 and 5 dB, which meets the literature (Hall and Haynes, 2001). Tinnitus is frequently reported as being extremely loud, but when matched with calibrated acoustic signals, is typically within 10 dB of the audiometric threshold (Hall and Haynes, 2001). More than half of our tinnitus participants have a central location (52.4%), which is in line with the finding reported in the literature (Pan et al., 2009).

Regarding Feldmann’s curves, the convergent (47.6%) and distant types (29.8%) are the most frequent, considering the sloping configuration in our sample. Our results are in accordance with the studies conducted by Goldstein and Shulman (2007). The most frequent residual inhibition type was negative (43.9%). Partial-to-complete residual inhibition was reported by 52.5%, which is far from that found in several studies in the literature that showed about 80% of participants with tinnitus reported some degree of RI. This can be explained by differences in the intensity, duration, and spectrum of the sound used to induce RI (Galazyuk et al., 2019). The duration of RI varies considerably among participants, ranging from several seconds to hours, scaling logarithmically with the duration of the preceding masking sound (Hazell and Wood, 1981; Terry et al., 1983).

Regarding the severity of tinnitus, participants with an abrupt tinnitus onset were more likely to have at least moderate tinnitus. This immediate interpretation of the result is that people who have a gradual tinnitus onset develop natural habituation processes effortlessly (Hallam et al., 1984). In analogy with the sensation of pain and phantom limb perception, tinnitus emerges from damages in the cochlea (e.g., hair cell loss or synaptic damages), leading to a frequency-specific decrease in electric output toward the brain. Our clinical data show that participants with tinnitus in this age group, with or without HL, have higher hearing thresholds, and interestingly that participants with moderate and severe hyperacusis have more risk of at least moderate tinnitus. Data from the literature indicate that there are common pathways for the pathophysiology of tinnitus and hyperacusis, resulting in a central compensatory gain due to reduced neural activity from a damaged cochlea (Knipper et al., 2013; Auerbach et al., 2014).

## Conclusion

Our study confirms that in older people, tinnitus is positively associated with HL and noise exposure. Indeed, HL and noise exposure are risk factors for tinnitus.

Nowadays, tinnitus is considered a symptom involving a network of peripheral and central pathways of the nervous system. Due to its complex nature, tinnitus should be approached in a multidisciplinary fashion involving various health professionals specialized in dealing with each of the dimensions encompassed within this symptom (Hall et al., 2018).

Our study puts in evidence some interesting findings, especially concerning audiological tinnitus characteristics or its development. Our data may contribute to defining the patient’s odds of developing a severe or catastrophic grade of tinnitus.

It was in the ABRs that we obtained the most exciting and promising results, namely, in the diminished I-III interval in participants without HL and no tinnitus compared to participants without HL and tinnitus, and the reduction of the amplitude in wave I in tinnitus participants compared with participants without tinnitus. Also, the increased amplitude of wave I has a protective factor to the odds of having tinnitus. Conversely, the increased ratio of V/I showed higher odds of developing at least moderate tinnitus. It should also be noted that there is a common denominator in all the findings in ABR, which is wave I. Future studies should be carried out with the main target of studying wave I in participants with tinnitus. If confirmed in more extensive population studies, these findings may be candidates as audiological biomarkers of tinnitus severity/presence. These are, indeed, the most original contributions of this study, since we have documented the relevant audiological tinnitus severity biomarkers.

Regarding DPOAE, findings highlight the correlation between HL and tinnitus. We can say that participants with tinnitus and relevant noise exposure have lower DPOAE between 500 and 8,000 Hz than participants without tinnitus, and participants with higher DPOAE have a lower risk of developing tinnitus.

Lastly, participants who had abrupt tinnitus onset and moderate or severe hyperacusis featured higher odds of at least moderate tinnitus.

Notable highlights of our findings that could serve as potential audiological biomarkers, in particular, wave I amplitude, wave I absolute latency, and interwave latency interval I-III, suggest the necessity to have appropriate tinnitus subtyping to understand the most probable underlying mechanisms and consequently the most appropriate diagnosis and treatment strategies.

Future research should be designed to improve the sensitivity of non-invasive electrophysiological measures of cochlear synaptopathy in humans and examine the broader neurophysiological impacts of noise exposure and devise a clear distinction between mechanisms more specific to tinnitus or HL. Advancing knowledge concerning potential tinnitus audiological biomarkers can be crucial for the adequate diagnosis and treatment of tinnitus.

## Data availability statement

The original contributions presented in this study are included in the article/Supplementary material, further inquiries can be directed to the corresponding author.

## Ethics statement

The studies involving human participants were reviewed and approved by Ethical Committee of CUF Infante Santo Hospital (26th November 2014), NOVA Medical School (no 65/2014/CEFCM). The patients/participants provided their written informed consent to participate in this study.

## Author contributions

HH conceived and designed this study and had contributions to all its stages. IP and AP performed the statistical analysis. HH, DR, SR, DH, NT, LB, and HC contributed equally to all other stages of the manuscript development, drafted, and revised the manuscript. DR worked with HH on the interpretation of results and created appendices. DR created all figures, abbreviations, references, and conceived the final version of the manuscript. JP, NT, and DH provided consultative advice and revised the final manuscript. All authors contributed to the article and approved the submitted version.

## Abbreviations

THI: Tinnitus Handicap Inventory
AEP: Auditory Evoked Potentials
ABR: Auditory Brainstem Response
OAE: Otoacoustic Emissions
DPOAE: Distortion Product Otoacoustic Emissions
OHC: Outer Hair Cells
HL: Hearing Loss
PTA: Pure Tone Average
HF_PTA: High-Frequency Pure Tone Average
ANFs: Auditory Nerve Fibers.
