# Supplementary material for: Audiological biomarkers of tinnitus in an older Portuguese population
Source: Front Aging Neurosci. 2022 Aug 24;14:933117. doi: 10.3389/fnagi.2022.933117 (PMC9449802; doi:10.3389/fnagi.2022.933117)
Supplement: Supplementary file 1 [file Table_1.DOCX]

Supplementary Material

**Supplementary Material 1.** Distribution and univariable analysis: comorbidities variables according to the presence of tinnitus. Legend. ^(1)^ Fisher's exact test**;** remaining p-values were obtained by Chi-Square test.

| **Variables** | **All patients (n=122)** | **With Tinnitus (n=92;75.4%)** | **Without Tinnitus (n=30;24.6%)** | ***p-*value** |
| --- | --- | --- | --- | --- |
| Diabetes | 15(12.3%) | 9 (9.8%) | 6 (20.0%) | 0.139 |
| Cardiovascular disease | 7 (5.7%) | 5 (5.4%) | 2 (9.1%) | 0.801 |
| Hypertension | 57 (46.7%) | 41 (44.6%) | 16 (53.3%) | 0.403 |
| Cholesterol | 70 (57.4%) | 52 (56.5%) | 18 (60.0%) | 0.738 |
| Thyroid problems | 12 (9.8%) | 11 (12%) | 1 (3.3%) | 0.168 |
| Measles n=121 | 92 (75.4%) | 67 (72.8%) | 25 (86.2%) | 0.141 |
| Meningitis | 3 (2.5%) | 3 (3.3%) | 0 | - |
| Mumps n=121 | 63 (51.6%) | 51 (56.0%) | 12 (40.0%) | 0.127 |
| Tuberculosis | 4 (3.3%) | 3 (3.3%) | 1 (3.3%) | 0.985 |
| Smoking habits | 53 (43.4%) | 42 (45.7%) | 11 (36.7%) | 0.389 |
| Familiar history with Tinnittus n=113 | 30 (24.6%) | 25 (29.4%) | 5 (17.9%) | 0.230 |
| Dizziness n=110 | 45 (36.9%) | 35 (41.2%) | 10 (40.0%) | 0.916 |
| Lesser noise tolerance n=104 | 55 (52.9%) | 45 (50.6%) | 14 (60.9%) | 0.377 |
| With deafness | 54 (47.4%) | 49 (53.3%) | 8 (26.7%) | **0.011*** |
| Noise exposition n=121  Non exposed  Exposed without protection  Exposed with protection | 75 (66.4%)  34 (30.1%)  4 (3.5%) | 56 (61.5%)  31 (34.1%)  4 (4.4%) | 24 (80.0%)  4 (13.3%)  2 (6.7%) | 0.093^(1)^ |
